# Genu Valgum.—II

**Published:** 1891-10-03

**Authors:** 


### SURGERY.

### GENU VALGUM.—II.

We take it that there is unanimous consensus of opinion that if non-operative measures may possibly suffice, that that plan of treatment should be adopted.

In a young child with a slight deformity it is essential that he should have a well-padded splint extending from the trochanter to six inches below the sole of the foot, applied firmly yet comfortably to the outside of the limb, in order to keep him off his feet. This should be worn by day only at first, but as soon as the child has become accustomed to its presence it could be worn at night as well. Every morning and two or three times in the day it should be removed, and the limb well bathed and rubbed, to improve the circulation and keep up the tone of the muscles; and the knee should be extended and moulded into position with as much force as the child can well bear. It is, of course, absolutely essential that the surgeon himself should give personal instruction and demonstration of these details, and from time to time see that the personal attendant of the child has not forgotten the proper manner of performing the manipulations.

At the same time the general health of the little patient should never be allowed to suffer; he should be carried, or wheeled in a perambulator, in the fresh air and sunshine; his diet should be carefully attended to; and tonics prescribed in whatever form may be for the time deemed necessary.

It is surprising how, in a few months of persistent treatment in this manner, a deformity will disappear; but it is then that even longer vigilance must be observed, lest the child be allowed too much freedom, and a disappointing relapse be the result. A short outside splint, or if it can be afforded, a light iron appliance, should be worn for a year or more, until the joint has become accustomed to its new position, and the bones have become too rigid to yield.

If the deformity be at all severe, it will be found impossible to keep an ordinary outside splint in position, for it will slip round towards the front of the limb, however carefully it may be bandaged. We are afraid that it is in such cases that splinting falls into disrepute, for after, may be, many months of patience bestowed, no benefit can be seen, and the friends relax their care and vigilance, and the child is allowed to run about, and very possibly become more deformed. In such cases it is well to have a lead or zinc splint that can be moulded to the outside surface of the limb, and straightened gradually as the deformity is lessened by the rest and repeated manipulations.

Where expense is no object, we have found that a double mechanical appliance applied to the outside of each limb, joined by a belt at the waist, and fastened to the boots, has given sufficient support to apply lateral pressure to the knees by means of a properly made pad, and has led to excellent results; and we have found that a child so treated may be allowed to walk at an earlier date in the treatment with impunity.

T. S. Ellis is a strong advocate for properly-applied calisthenics in the treatment of knock-knee, and in a paper published in the *Medical Journal* of 1888 he demonstrates how the muscles, reaching from the pelvis to the knee, in the action of raising the weight of the body on tiptoe, do tend to pull the knees *outward*, and he therefore strongly urges the necessity of exercises tending to the development of this force, such as pulling a weight suspended over a pulley; or even of insisting upon the patient getting into the habit of merely raising himself on his toes repeatedly. He says he has found good results in various degrees of this deformity, and, furthermore, denounces osteotomies and resections as unwarrantable mutilations. Though we have seen cases in which, we fear, such treatment would have been of no avail, we must say it is well worthy of trial.

We now come to those cases in which operative treatment is the only one to be adopted if we wish the child to be turned out in the world with a straight pair of legs; and, before going further, we should like to say that in operating upon any of these cases we have merely put the limb in good position, and we must not lose sight of the very important fact that it is necessary to keep it so, and it by no means follows because a patient is wearing irons after such an operation that the procedure has been a failure. It is as absolute and essential a portion of the treatment as the operation itself.

After a careful study of the authorities on the subject, we should consider operation advisable in the following cases: 1. Moderate cases in early childhood, where it is quite impossible to have instructions carried out from persistent neglect on the part of the child's parents. 2. Severe cases at all ages. 3. Moderate cases after the age of ten, if the parents desire it.

In double *genu valgum* in a child 4 inches would be a moderate case, 5 inches fairly severe, and 10 inches an extreme case.

There are two divisions of operations, viz., forcible straightening (*redressement*) and osteotomy.

Forcible straightening seems to be so severe a measure and so lacking in precision and nicety, that we have no hesitation in this article in condemning it.

Osteotomy of the lower end of the femur with Ogston for its pioneer and Macewen as improver, has, perhaps, been one of the most popular operations of recent years; but for its advent, numberless cases of this unsightly deformity would still be found in our midst.

With commendable caution, however, Edmund Owen, in the Harveian lectures of 1880, said: "I suppose that no new operation has ever met with so cordial a reception and so general an adoption as that of osteotomy; and as regards its performance, of inestimable value, doubtless, in certain cases of knock knee and bandy leg, I apprehend that the only reason for its becoming less in surgical fashion will be the fact of there being no more rickety limbs discoverable; certainly in the visionary 'City of Health' the osteotomist will find his occupation almost gone. In the early days of a new operation it is competent for every surgeon to hold his own opinion concerning it; and for my own part, I cannot but think that, in an enthusiastic adoption of mild or heroic cutting operations in dealing with these numberless ill-shapen little limbs, one is apt to lose sight of grand and conservative principles."

He insists upon careful after-treatment, remarking that it is one thing to see a case straight from the osteotomist's hands and another to see it a few years afterwards.

In selecting the operation to be done, it is well to be guided by the dictum of an old surgeon, "Do the operation which in your hands has proved the most successful."

The osteotomy originated by Ogston, with its one grave fault of opening the knee joint, has now been generally discarded in favour of one or other of the supracondyloid sections, the one invented by Macewen being deservedly the most popular. A detailed description of these various procedures is not here called for; suffice it to say that the most rigorous antiseptic precautions should be observed, and that in an ideal case no subsequent dressing should be necessary.

The limb should be put up in a position, slightly bowed, if anything, at the end of a fortnight in plaster of Paris, and kept so encased for another month or six weeks; and the patient should not be allowed to walk about without the application of an outside splint, or, better still, a well-made mechanical appliance, which should be worn for many months. The younger the patient, the longer should the artificial support be applied.

In the family practices of the busy practitioners of our large towns, cases of knock knee must of necessity be of frequent occurrence, and in the early detection of them they have the opportunity, by careful and painstaking treatment, of avoiding more serious trouble and anxiety in after years, and it behoves them, under such circumstances, not to be led into the far too easy expression of opinion that "the child will grow out of it."

